# A Nine-Year Follow-Up of Stage II Preiser’s Disease Treated With a Temporary Dorsal Wrist-Spanning Plate: A Case Report

**DOI:** 10.7759/cureus.46474

**Published:** 2023-10-04

**Authors:** Margaret Pennington, Molly Milano, Daniel Fletcher, Moody Kwok, William Emper, Pedro Beredjiklian, Jack Abboudi

**Affiliations:** 1 Division of Hand Surgery, Rothman Orthopaedic Institute, Philadelphia, USA; 2 Division of Sports Medicine, Rothman Orthopaedic Institute, Philadelphia, USA

**Keywords:** orthopedic hand surgery, bridge plate fixation, joint offloading, avascular necrosis scaphoid, preiser’s disease

## Abstract

Preiser’s disease, also known as avascular necrosis of the scaphoid, is a rare condition that is incompletely understood in regard to pathophysiology, diagnosis, and management. There have been numerous case reports and case series evaluating a variety of conservative and operative interventions, but optimal treatment has not been well established. We describe the case of a 20-year-old female with stage II Preiser’s disease that was managed with a vascularized bone graft from the 1,2 intercompartmental supraretinacular artery, in addition to temporary dorsal wrist-spanning bridge plate fixation. At the nine-year follow-up, the patient had near full wrist range of motion, no pain, and radiographs showing preserved carpal alignment and a scapholunate angle within normal range. Our findings suggest that this surgical technique is a viable option for restoring scaphoid vascularity, preserving carpal alignment, and halting disease progression.

## Introduction

Preiser’s disease is characterized by avascular necrosis of the scaphoid that is thought to be due to a reduction in the dorsal blood supply leading to compromised vascularity of the proximal two-thirds of the bone [1]. It most commonly affects females, and the age at the time of diagnosis ranges from 18 to 70 years with an average age of 46 years [1,2]. The etiology of the disruption of blood flow to the scaphoid remains unclear, with several proposed theories, including trauma, steroid use, chemotherapy, connective tissue disorders, congenital scaphoid hypoplasia, smoking, and alcohol intake [3-5].

Due to the idiopathic nature of Presier’s disease and its rare occurrence, there are several proposed treatment options with no single gold standard [2,5]. Management options range from conservative measures to operative interventions, including, but not limited to, arthroscopic debridement, curettage and bone grafting, silicone replacement, proximal row carpectomy, four-corner fusion, wrist arthrodesis, and vascularized bone grafts (VBGs) to restore blood supply to the proximal two-thirds of the scaphoid [1,5]. The choice of treatment option depends on the stage of disease and the degree of degenerative changes in the carpal bones, with ultimate attention being paid to the integrity of the cartilage at the proximal pole of the scaphoid [1]. Patients with more advanced diseases have been shown to have improved pain scores with surgical versus nonoperative management [2]. The use of VBGs as a treatment option for Preiser’s disease is promising and has been shown to restore vascularity to the affected scaphoid, granted that protective postoperative steps are taken to ensure proper integration of the VBG [5]. There are no reported cases in the literature of the utilization of joint offloading procedures such as external fixation or dorsal wrist-spanning plate fixation as part of the surgical management for Preiser’s disease. However, there are numerous case reports and case studies of successfully employing this technique as an adjunct to treatment in Kienböck’s disease to decrease the force transmitted through the lunate during the revascularization process [6-9]. In this case report, we present the surgical technique with a nine-year follow-up of a 20-year-old female with Preiser’s disease who was treated with a VBG in addition to a temporary dorsal wrist-spanning plate fixation for mechanical joint offloading during integration of the VBG.

## Case presentation

A 20-year-old female presented to the office with an atraumatic onset of significant radial-sided right wrist pain for approximately three and a half months. She had moderate tenderness to palpation about the volar scaphoid and mild tenderness to palpation about the dorsal radial wrist. She endorsed pain with wrist range of motion and the examiner noted decreased grip strength in the right hand, likely secondary to pain. There was visible swelling about the volar wrist at the level of the flexor carpi radialis insertion. She had normal sensation and brisk capillary refill. Finklestein’s test and carpal instability with shift testing were negative, and the distal radioulnar joint was stable. A course of conservative management failed, including oral anti-inflammatory medications and a trial of a splint prescribed by urgent care.

**Figure 1:**
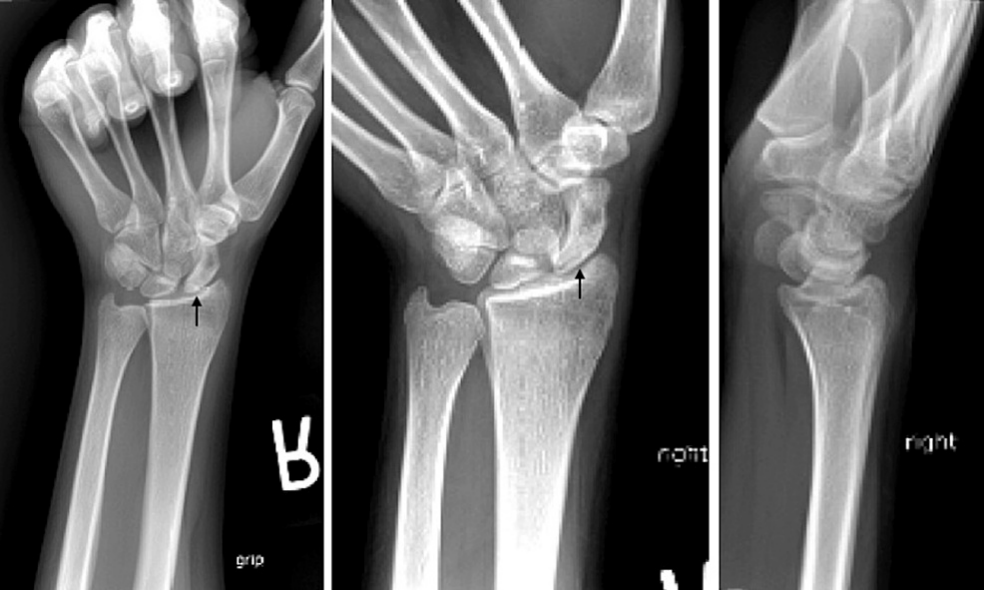
Initial X-rays of a 20-year-old female who presented with 3.5 months of radial-sided wrist pain. X-rays reveal sclerosis of the proximal scaphoid (black arrows) without evidence of carpal malalignment.

**Figure 2:**
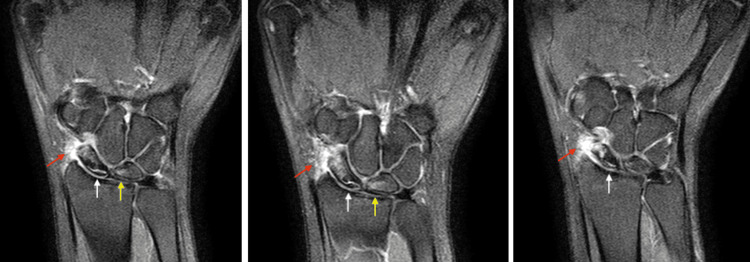
T2-weighted coronal images from MRI of the right wrist without contrast of a 20-year-old female who presented with 3.5 months of radial-sided wrist pain. MRI reveals avascular necrosis in the scaphoid extending from the waist to the proximal pole (white arrows), mild reactive changes in the lunate without collapse (yellow arrows), and a radial-sided wrist effusion (red arrows).

**Figure 3:**
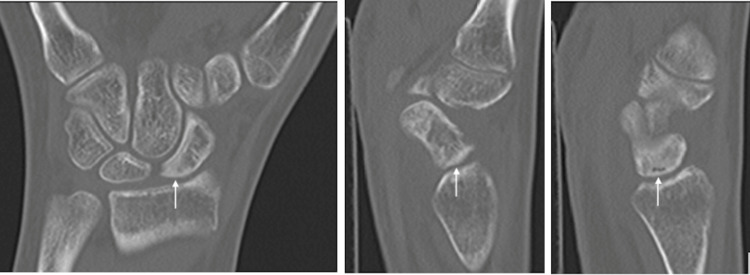
Coronal and sagittal CT images of the right wrist without contrast of a 20-year-old female who presents with 3.5 months of radial-sided wrist pain. CT demonstrates sclerosis of the proximal pole of the scaphoid (white arrows) without evidence of fragmentation, fracture, or carpal collapse.

X-rays demonstrated sclerosis of the proximal half of the scaphoid in addition to a mild flattening of the lunate particularly along its radial column (Figure 1). There was no radiographic evidence of degenerative changes or articular collapse. An MRI was obtained which demonstrated avascular changes in the proximal pole of the scaphoid with diffuse edema throughout the scaphoid and mild reactive changes of the neighboring lunate (Figure 2). A CT scan again revealed the avascular changes in the proximal pole in accordance with the previous MRI results and was unremarkable for any fragmentation or articular surface or articular segment collapse (Figure 3).

After imaging was obtained, the role of conservative management with continued observation, nonsteroidal anti-inflammatory drugs (NSAIDs), and splinting versus surgical intervention to restore blood supply to the scaphoid was discussed with the patient. Due to a lack of improvement with conservative management consisting of immobilization and NSAIDs before presentation, the patient elected to proceed with surgical intervention.

Surgery

A right wrist VBG was performed using the 1,2 intercompartmental supraretinacular artery (ICSRA) from the distal radius and a right dorsal wrist-spanning plate was applied to function as an internal-external fixator. An S-shape incision was made along the dorsal radial aspect of the wrist, beginning distally at the anatomic snuffbox and extending proximally along the fourth extensor compartment. A small transverse arthrotomy was made to access the scaphoid. The scaphoid and its articular surfaces were without any significant degenerative changes. A burr was utilized to open a 12 × 6 mm slot in the scaphoid in the proximal half of the bone dorsally. The VBG was then raised 15 mm proximal to the radiocarpal joint line and the pedicled flap appeared viable with appropriate bleeding surface. The VBG was placed deep into the second extensor compartment tendons and into the previously constructed slot in the scaphoid with no vessel kinking.

Following the VBG, a dorsal spanning wrist plate was then placed as an internal-external fixator equivalent to span the wrist and unload the scaphoid. The plate was fixed distally in the middle finger metacarpal and proximally in the radial shaft. Closure was performed in a standard fashion.

Postoperative course

**Figure 4:**
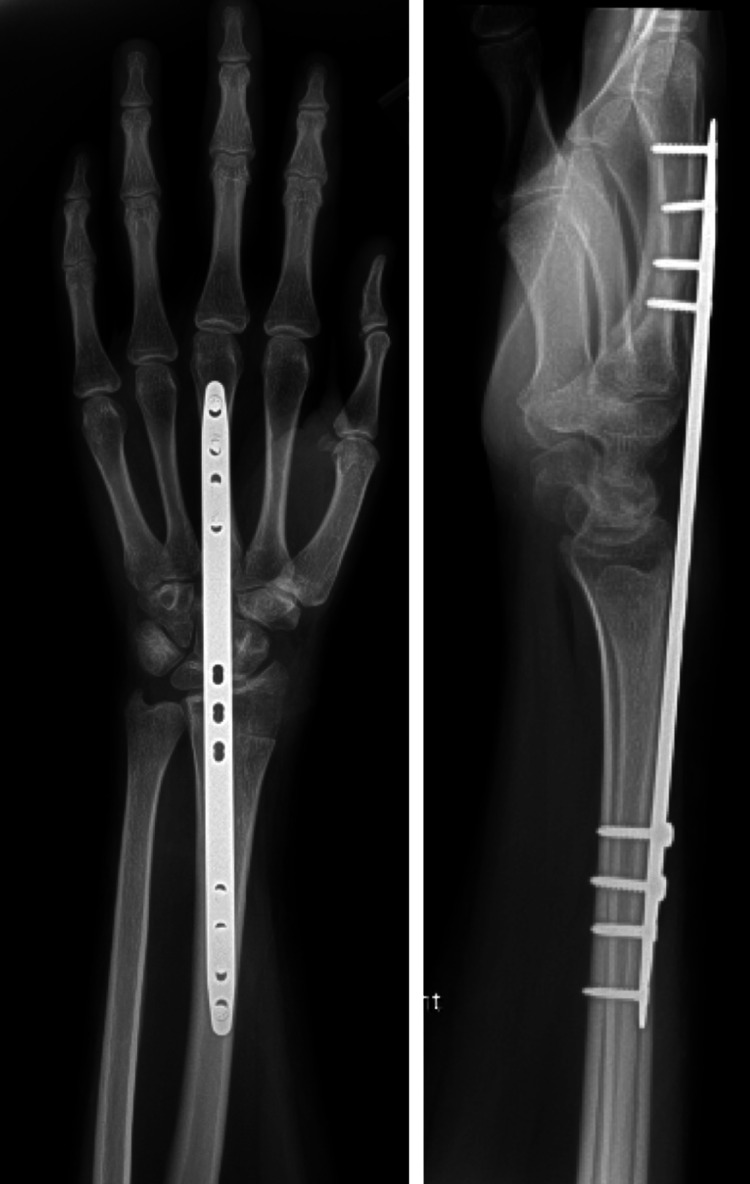
Postoperative X-rays following VBG from the 1,2 intercompartmental supraretinacular artery with a temporary dorsal spanning bridge plate.

Postoperatively the patient recovered well. Her incisions healed without complications. She wore a splint full-time for eight weeks and then transitioned to splint use only when out of her home. She worked with occupational therapy (OT) on wrist pronosupination and finger range of motion and was instructed to only use the right wrist for light activities. The dorsal spanning wrist plate was left in place for four months (Figure 4) and then removed without complication.

**Figure 5:**
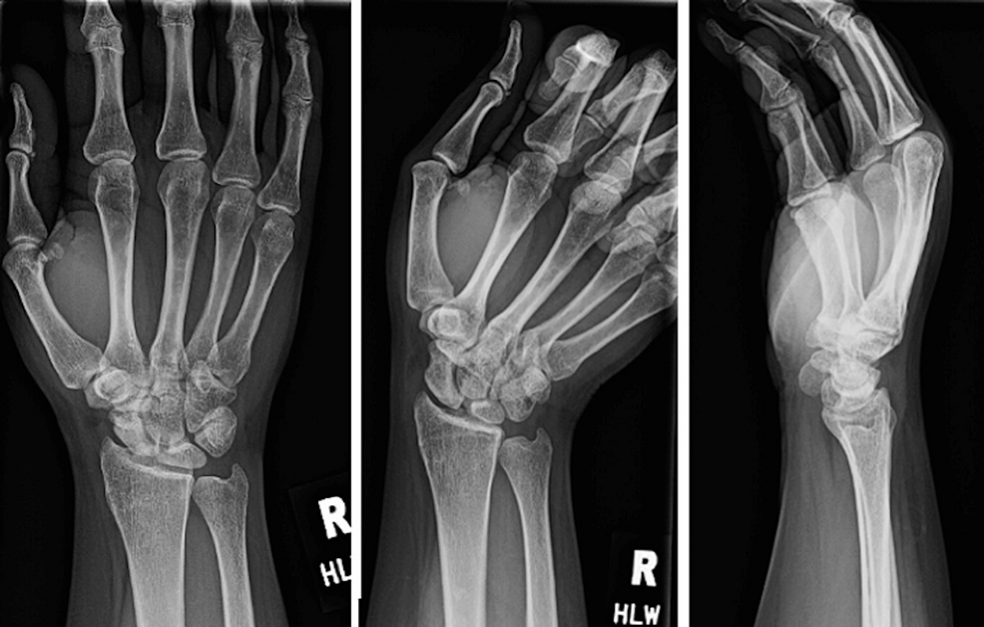
X-rays nine years postoperatively from VBG from the 1,2 intercompartmental supraretinacular artery with a temporary dorsal spanning bridge plate showing maintained carpal alignment and no further disease progression.

She continued working with OT after the removal of hardware to restore the wrist range of motion. In her postoperative visits following the removal of hardware, she had no wrist pain and was able to use her wrist to do activities of daily living. The patient was most recently seen at nine years postoperatively at which point she had no wrist pain and continued to use her operative hand without limitations. Upon physical examination at this time, she had a near-full wrist range of motion with only a minor discrepancy compared to the contralateral side. X-rays at nine years postoperatively showed maintained carpal alignment with a scapholunate angle of 60 degrees and with no further disease progression or fragmentation of the scaphoid (Figure 5).

## Discussion

We present the case of a 20-year-old woman with stage II Preiser’s disease who was managed with a VBG from the 1,2 ICSRA in addition to a temporary dorsal wrist-spanning bridge plate.

Since the initial documentation of Preiser’s disease in 1910, the condition’s rarity has led to challenges in studying it from an epidemiological perspective, understanding its natural history, and assessing outcomes following various proposed surgical management choices [3]. The typical presentation of a patient with Preiser’s disease is radial-sided wrist pain, tenderness to palpation about the scaphoid, and diminished wrist range of motion [3]. The diagnosis can be confirmed with radiographs and advanced imaging which show scaphoid sclerosis on radiographs and MRI findings of signal changes within the scaphoid bone, most commonly in the proximal pole. Late MRI findings include cystic changes within the scaphoid and fragmentation and/or fracture within the bone [3].

The management of Preiser’s disease depends on its classification which is based on radiographs. There have been four proposed stages in the original classification described by Herbert and Lanzetta in 1994, including (I) normal X-rays with a positive bone scan, (II) increased density of the proximal pole of the scaphoid, (III) fragmentation of the proximal pole of the scaphoid with/without pathologic fracture, and (IV) carpal collapse [10]. Recommended treatment for stage I includes conservative management, with options including immobilization, NSAIDs, and physiotherapy. However, in a systematic review comparing these conservative modalities, Kazemi et al. found that none of these measures were effective in slowing disease progression and suggested that there may be an advantageous role of surgery even in early disease stages [2]. Treatment options for stages II through IV include a variety of surgical interventions. Proposed operative options for early radiographic stages of Preiser’s disease include arthroscopic scaphoid debridement, VBG, curettage and bone grafting, arthroscopic drilling, and closing wedge radial osteotomies. Recommended treatment options for advanced radiographic stages of Preiser’s include proximal row carpectomy (PRC), scaphoid excision with four-corner fusion, silastic implants, and wrist arthrodesis [1-3]. A systematic review found that VBG is the most effective treatment in terms of pain relief, improvement in range of motion, and reversing disease progression for stage II Preiser’s disease [2]. In stage III Preiser’s, the same systematic review found that PRC was the most commonly used and most effective treatment in terms of pain relief and range of motion improvement [2]. Optimal treatment management in stage IV is unclear.

Although Preiser’s disease is a rare entity, numerous case reports and case series have been published evaluating outcomes after conservative and operative management of this disease. After the first description of a VBG for treatment of scaphoid avascular necrosis in 2000, several case series have been performed evaluating outcomes of this specific technique which show improvement in pain and range of motion postoperatively [4,5,11]. However, most of these studies are limited to short-term follow-ups and specific operative techniques.

The case report we present is the first report to our knowledge that evaluates the outcome after operative management with a combination of a VBG from the 1,2 ICSRA with a temporary dorsal wrist-spanning bridge plate. While joint offloading procedures such as external fixation or dorsal wrist-spanning plate fixation have been described for a similar pathology in Kienböck’s disease, these same techniques have not been described as an adjunct in the management of Preiser’s disease [6-9]. The theoretical advantage of adding a temporary dorsal spanning bridge plate is additional offloading of the radiocarpal joint and wrist stabilization during the integration stage of the VBG. While there are a variety of treatment options for patients with a diagnosis of Preiser’s disease, the patient we presented is at the youngest end of the spectrum and therefore every effort should be made to retain normal carpal kinematics with joint preserving interventions. The promising nine-year results of this case report suggest that a combination of a temporary dorsal spanning bridge plate with VBG should be considered as an operative option in young patients with stage II Preiser’s disease.

The main limitation of this case report is that the patient in our study was a young patient with good innate healing capabilities. While the age range affected by Preiser’s disease is variable, this technique may not be ideal for patients of all ages given that older patients often have less healing potential. In addition, as this is a single case report, subsequent case series utilizing plate offloading would provide more data on the advantages of this surgical technique.

## Conclusions

Preiser’s disease is a rare condition with a variety of proposed treatment options depending on the radiographic stage of the disease but without well-established guidelines on management strategies. The paucity of literature on outcomes after surgical management highlights the importance of publishing case reports and case series to add to the understanding of this condition. This case report describes the novel combined surgical technique of a VBG with a temporary dorsal spanning bridge plate. The successful results at the nine-year follow-up for this patient suggest that this is a promising management option for the operative treatment of Preiser’s disease.
